# EST2uni: an open, parallel tool for automated EST analysis and database creation, with a data mining web interface and microarray expression data integration

**DOI:** 10.1186/1471-2105-9-5

**Published:** 2008-01-07

**Authors:** Javier Forment, Francisco Gilabert, Antonio Robles, Vicente Conejero, Fernando Nuez, Jose M Blanca

**Affiliations:** 1 Instituto de Biología Molecular y Celular de Plantas (IBMCP), Universidad Politécnica de Valencia – Consejo Superior de Investigaciones Científicas, Avenida de los Naranjos s/n, 46022 Valencia, Spain; 2 Grupo de Arquitecturas Paralelas (GAP), Universidad Politécnica de Valencia, Avenida de los Naranjos s/n, 46022 Valencia, Spain; 3 Centro para la Conservación y Mejora de la Agrodiversidad Valenciana (COMAV), Universidad Politécnica de Valencia, Avenida de los Naranjos s/n, 46022 Valencia, Spain

## Abstract

**Background:**

Expressed sequence tag (EST) collections are composed of a high number of single-pass, redundant, partial sequences, which need to be processed, clustered, and annotated to remove low-quality and vector regions, eliminate redundancy and sequencing errors, and provide biologically relevant information. In order to provide a suitable way of performing the different steps in the analysis of the ESTs, flexible computation pipelines adapted to the local needs of specific EST projects have to be developed. Furthermore, EST collections must be stored in highly structured relational databases available to researchers through user-friendly interfaces which allow efficient and complex data mining, thus offering maximum capabilities for their full exploitation.

**Results:**

We have created EST2uni, an integrated, highly-configurable EST analysis pipeline and data mining software package that automates the pre-processing, clustering, annotation, database creation, and data mining of EST collections. The pipeline uses standard EST analysis tools and the software has a modular design to facilitate the addition of new analytical methods and their configuration. Currently implemented analyses include functional and structural annotation, SNP and microsatellite discovery, integration of previously known genetic marker data and gene expression results, and assistance in cDNA microarray design. It can be run in parallel in a PC cluster in order to reduce the time necessary for the analysis. It also creates a web site linked to the database, showing collection statistics, with complex query capabilities and tools for data mining and retrieval.

**Conclusion:**

The software package presented here provides an efficient and complete bioinformatics tool for the management of EST collections which is very easy to adapt to the local needs of different EST projects. The code is freely available under the GPL license and can be obtained at . This site also provides detailed instructions for installation and configuration of the software package. The code is under active development to incorporate new analyses, methods, and algorithms as they are released by the bioinformatics community.

## Background

Recent advances in high-throughput sequencing technology have provided a mechanism to gain genomics insight on species without a complete genome sequence by generating expressed sequence tags collections (ESTs, [1]). ESTs are single-pass, partial sequences obtained from randomly selected complementary DNA (cDNA) clones and need to be processed and annotated to provide a biologically relevant data set. They include low-quality and vector regions that must be identified and removed to obtain high-quality, clean sequences suitable for further analysis. In addition, due to the random selection of the sequenced cDNA clones, a clustering step is needed to obtain a non-redundant set of unique consensus sequences, or unigenes. Finally, functional and structural annotation of the unigenes is required in order to add relevant biological information to the sequences. All these data must be conveniently stored and organized in a structured database, and interfaces must be set up for end-users to efficiently retrieve and mine all these data.

Due to the high number of sequences in most ESTs datasets, different computer-based methods are required to process, annotate, record, display, and retrieve the data. These methods are applied sequentially from the input raw sequence data to the final searchable, fully annotated EST database, and knowledge of computing science is needed to arrange them in an efficient and reliable analysis pipeline. The usual approach to this problem is to build an in-house prepared set of script programs that semi-automates the analysis. This solution requires highly skilled bioinformatics staff capable of programming and using the scripts, and is inefficient because a different pipeline should be prepared for each project, and because the resulting semi-automated process is difficult to maintain and lacks reproducibility. It would therefore be more convenient to use a well tested, freely available automatic tool. In our opinion, this application should ideally have the following features: 1) to be fully automated in a pipeline covering all the steps from the input chromatogram files to a clean, annotated web-searchable EST database, 2) to be highly modular and adaptable, 3) to be able to run in parallel in a personal computer (PC) cluster, thus benefiting from the multiprocessing capabilities of these systems, 4) to use third-party freely-available programs, in order to ease the incorporation of the improvements made by others programmers, 5) to include a highly-configurable and extensible user-friendly interface to perform data mining by combining any search criteria, fitting the final user needs, and 6) to be based on an open source license to allow a continuous development by a community of users and programmers, as well as its customization for the particular needs of different projects.

Some applications, including PipeOnLine [2], ESTAP [3], PartiGene [4], ESTIMA [5], EST-PAGE [6], ParPEST [7], GARSA [8], or openSputnik [9], have been proposed and they fulfill a certain number of the desired characteristics. However, as far as we know, none of these packages are endowed with all the requirements indicated above, especially the code availability, enabling costumization, and the automatic creation of a user-friendly web site to perform complex queries ready to be deployed in a production environment.

In an attempt to fulfill the need for an analysis software which accomplishes all the mentioned requirements, we have developed a software package, namely EST2uni (EST analysis software TO create an annotated UNIgene database). This pipeline has been tested through three genomics projects which we are involved in: Citrus Functional Genomics Project [10], ChillPeach Project [11], and Spanish Melon Genomics Project [12]. EST2uni uses a set of chromatogram files as input to produce a structured and annotated EST database, as well as a web site to perform complex queries and data mining. Configuration of the pipeline is done by just editing a single well-documented text file. After initial set up configuration, the pipeline is completely automated, and can be run in parallel in a PC cluster using the load distribution tool CONDOR [13]. Its modular structure provides an easy way to adapt the analysis to the special requirements of individual projects. Furthermore, the code is designed to easily integrate well-tested, widely-used, freely available third-party tools, either as locally installed programs (e.g., Primer3 [14]) or as web services (e.g., GEPAS [15] and Babelomics suites [16]). The software package is freely distributed [17] under a GPL license, and can be easily installed in a standard Linux system running Apache HTTP Server, Perl scripting language, MySQL database management system, and PHP language.

## Implementation

The EST2uni package consists of: 1) a set of Perl modules that perform both the EST analysis and the database creation, 2) a set of PHP scripts to generate the browseable database-interacting web pages, and 3) a set of PHP modules with the functions called by the PHP scripts. The EST analyses are performed by the main Perl script, which manages the execution of several third-party, freely-available, standard tools commonly used for EST analysis, as well as a number of home-made analyses. The running parameters used by the pipeline and the external tools are stored in a well-documented, plain text configuration file which can be modified with a text editor. This configuration file contains all the required information to run the pipeline, like file paths, analyses to be performed, and paths and parameters for the programs installed to run the analyses. The software package is distributed with a single minimal-analysis example configuration file where default parameters used by EST2uni and external tools can be inspected and modified if desired. Running parameters considered as default ones by developers of the external tools used have been selected as a starting point in this example configuration file. Each annotation analysis is controlled by a different Perl module, and the results from different analyses are stored at independent tables in the database to facilitate the future addition of new methods and analyses. As a consequence, all analyses can be run independently at different times, and the annotation modules could be re-run at any time with updated external databases. Parallelization is also managed by an independent Perl module which splits the analysis in small tasks, manages their execution in different nodes, and join the results, using the load distribution tool Condor, as explained above. It should however be easy to hack the code in order to use a different load distribution tool.

The package is distributed with a complete working web site built by EST2uni using PHP. The visual design is controlled by using CSS, and special attention has been paid to the modularity of the PHP code, so that it is very easy to add new functionalities to the web pages and to customize their appearance. This adaptation should be easily performed by any project administrator because the PHP code appearing on the browseable web pages is minimal (e.g., the queries page has just 14 PHP lines). MySQL authentication can be used to maintain the privacy of the data of the different projects.

## Results

### EST analysis

**Figure 1:**
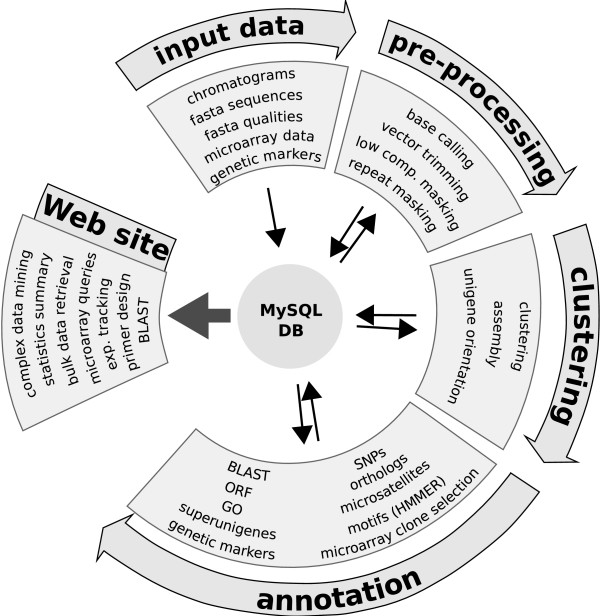
**Diagram of the EST2uni processing pipeline**. Pipeline showing the different analyses performed by EST2uni.

A standard EST analysis pipeline includes the following steps: 1) EST pre-processing, 2) EST assembly, and 3) functional and structural annotation of the resulting unigenes. Below we describe the analysis and methods currently implemented in EST2uni (Figure 1). However, the modular design of the package makes it easy to include new analyses. Furthermore, it is possible to run only specific parts of the pipeline just by indicating it either in the configuration file or in the command line. Next, we describe the steps followed by the EST analysis pipeline.

#### Pre-processing

Pre-processing is the first step performed, and includes base calling, vector and low-quality regions removal, masking of repeats and low-complexity regions, and contaminant sequence detection and removal. The result is the generation of a clean, high-quality EST sequence set. Both chromatogram and FASTA sequence files with or without quality scores are accepted as entry point to the analysis. Base calling and quality score assignment from chromatograms are performed with phred [18]. Low quality and cloning vector regions are removed from the sequences with Lucy [19], and repetitive elements and low complexity regions are masked with RepeatMasker [20] and seqclean [21], respectively. For repeat masking, a taxon-specific repeats database can be used. Unexpected vector sequences, probably coming from contamination by manipulation at the laboratory, are also removed with seqclean, using NCBI's UniVec database [22].

#### Assembly

Clean, high-quality EST sequences obtained in the pre-processing step are assembled in contigs and singletons in order to eliminate sequence redundancy and a non-redundant unigene set is obtained. Either CAP3 [23] or TGICL [21] can be used for the assembly step, and consensus sequences for the contigs are determined using the quality scores of the individual ESTs to resolve sequence discrepancies. A set of unigene clusters (or "superunigenes"), grouping different unigenes with extensive sequence overlapping, can also be obtained. These superunigenes could represent gene families, alternative splicing or polymorphism. Poly(A/T) tails and open reading frames (ORFs), predicted using ESTScan [24], are used to reverse the sequences when necessary.

To assist in the design of a cDNA microarray derived from the EST collection, one cDNA can be selected as the best representative for each unigene or superunigene. The criteria to choose this cDNA are based on a number of user-defined restrictions (extensive overlapping with the unigene consensus sequence, EST sequence length above a given threshold, GC content, etc.) and/or preferences (e.g., the most 5' or 3' clone, or the longest or shortest one, etc.). When no clone in an unigene satisfies all the above mentioned restrictions, these can be progressively relaxed until a representative is selected. Since clones from different unigenes in a superunigene are very similar, they are expected to have the same mRNA targets under standard hybridization conditions when used as probes in cDNA microarrays [25], and superunigene representatives can also be selected to be printed in the microarray, which reduces spot cross-hybridizations.

#### Structural annotation

Unigenes can be annotated using different modules, following user specifications in the configuration file. SSR microsatellites can be detected with sputnik [26]. Putative single nucleotide polymorphisms (SNPs) can be found by using a locally developed algorithm. Since ESTs have frequent sequencing errors, only positions with a quality score above a user-specified threshold value are considered, and sequence discrepancies between ESTs in the same contig are marked as putative true SNPs only if the polymorphism is confirmed by more than one EST in the contig. When a file including a set of primer pairs is provided, in-silico PCR experiments can be performed with ipcress [27] to integrate information about existing PCR-based molecular markers and associate these markers to specific unigenes. RFLP information can also be integrated with the unigenes via the cDNA clones. An RFLP is a clone proven to be useful to identify differential hybridization band patterns when used as a probe against DNA samples coming from different sources and digested with an specific restriction enzyme. A file with RFLP names and corresponding cDNA clones and restriction enzymes can be used to annotate unigenes coming from these RFLP clones. Finally, when cDNA libraries are constructed using oligo-dT as a primer for the reverse transcriptase reaction, unigenes can be aligned with protein databases from species with a complete genome sequence to predict if there are full-length clones for each unigene.

#### Functional annotation

For functional annotation, different similarity search tools can be used. Unigene comparisons against a set of user-defined nucleotide and/or protein databases are carried out by BLAST [28] using the search parameters indicated in the configuration file. The descriptions of the BLAST hits obtained with the different BLAST runs are then parsed to yield a descriptive annotation for each unigene. Descriptions containing some word from a user-defined list including words as "unknown" or "hypothetical" are considered uninformative and unsuitable for annotation of the unigenes. The annotation is in the form "Similar to" or "Highly similar to", depending on the E value of the alignment with the corresponding BLAST hit. Gene Ontology classification (GO, [29]) can also be obtained from BLAST against a set of user-defined GO-annotated databases. A HMMER [30] search against a Pfam database [31] can be used to locally search protein domains in each unigene. A bi-directional BLAST comparison can also be performed with a number of user-defined species-specific sequence databases in order to obtain a set of putative orthologs. In this analysis, two sequences are considered orthologs when each one is the first hit in a BLAST search with the other. Finally, when EST collections can have contaminant sequences coming from species other than the intended one, e.g. fungus sequences in plant EST libraries made with infected plants, a BLAST analysis can be done using taxon-specific databases to predict the source organism and flag the putative contaminant sequences.

#### Microarray expression integration

EST2uni is ready to incorporate microarray expression data for each unigene. Normalized expression data coming from different arrays and experiments can be easily added to the database and referred to the corresponding unigenes. Database can also store details about biological samples, tissues and conditions used for each experiment, while referencial integrity is required to reproduce the standard work flux in microarray experiments. There are several ways of retrieving this expression data from the web site depending on the number of unigenes involved. A graphical representation of expression data in all the microarray experiments done is created for each unigene, which is accesible from the individual unigene web pages. Bulk data retrieval is also posible, so that expression data for all unigenes or for any set of unigenes obtained in a query can be easily obtained. Furthermore, this data can be directly sent to the GEPAS analysis suite [15] or downloaded as a text file ready to be used with the MeV software [32].

### Parallelization

Execution times in standard EST analysis pipelines are usually very long because of the high number of sequences to be processed and the big computational costs of some analyses performed. EST2uni can achieve a reduction in these execution times by parallelization, because it can be run either in sequential mode or in a parallel environment using a load distribution tool. When run in parallel mode, EST2uni divides the analysis in several tasks that are asigned to different processing nodes and manages the execution of these tasks with the aim of keeping the different nodes busy, thus taking maximum advantages of the processing resources available in the system. These tasks are created by splitting the set of sequences to analyze in several chunks, and running independent subanalysis in parallel. EST2uni sends these jobs to the load distribution tool cue and checks that they run as expected. The pipeline is distributed with the code necessary to use CONDOR [13] as the load distribution tool. However, the high modularity of the code makes it easy to adapt EST2uni to use another load distribution tools, like openMosix [33] or openPBS [34].

**Figure 2:**
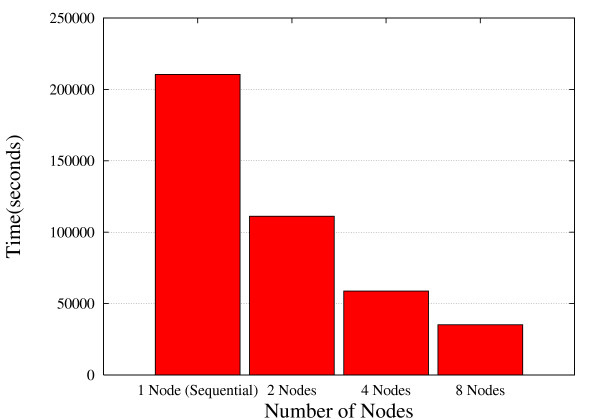
**Parallelization efficiency using different numbers of computer nodes**. Time required to perform the complete analysis of 8,000 ESTs using EST2uni in sequential mode and in parallel mode with 2, 4 or 8 biprocessor computer nodes.

Although some of the work load is done by third-party tools with its own parallel implementations (i.e. Blast, HMMER), we decided to use this EST2uni-driven unique model of parallelization for the whole because this approach enables to run in parallel even those analysis tools which are not designed to be run in that way. Moreover, because the different parallel tools have different parallelization infrastructures and requirements, the parallel code implemented in EST2uni is directly applied to their sequential implementations, so avoiding use of the parallel versions. Anyway, if a load distribution tool cannot be installed, the pipeline is also able to take advantage of parallelization by using the parallel versions of the third-party capable software like mpiBLAST [35] or HMMER using pvm (Parallel Virtual Machine, [36]). This optimal use of the available resources significantly reduces the time required to complete an EST analysis. Figure 2 compares the time required by EST2uni to completely perform the analysis of 8,000 ESTs in sequential mode (a single biprocessor node is used) with respect to the time required in parallel mode when using 2, 4, and 8 biprocessor nodes. Clearly, the time is considerably reduced when the number of nodes is increased. When 2 nodes are used, the time required is 1.9 times lower than when the analysis is performed in sequential mode. This time is 3.6 and 6.22 times lower when using 4 and 8 nodes, respectively. This represents an almost linear reduction in the processing time as the number of nodes grows, which shows the high scalability achieved by the EST2uni application.

**Figure 3:**
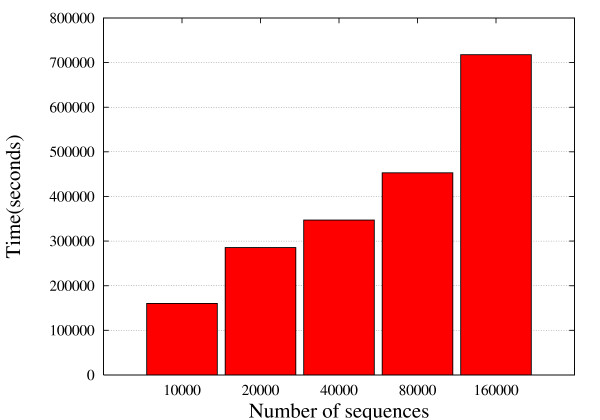
**Parallelization efficiency using different numbers of input ESTs**. Time required to perform a complete analysis of 10,000, 20,000, 40,000, 80,000, and 160,000 ESTs using EST2uni in parallel mode with 8 biprocessor computer nodes.

Regarding the ability of EST2uni to properly manage parallelization in the analysis of big EST collections, Figure 3 shows the time required to finalize a complete analysis of different numbers of input EST sequences using the pipeline in parallel mode with 8 biprocessor nodes. A set of 10,000 ESTs were completely processed, assembled, and annotated in two days, and more than 150,000 ESTs in just about one week, showing the robustness of the parallelization implemented. Notice that while the number of input ESTs increases by a factor of 16, the time needed to complete the full analysis increases only by a factor of 4.5. The main reason for this is that some analyses are performed on unigenes rather than ESTs, and unigene number is not increased by the same factor than EST number due to higher redundancy of bigger EST collections. It should be noted, however, that an important part of the parallelization performed by EST2uni is done before unigenes assembly, which highly contributes in that time reduction.

### Database

EST2uni creates and populates a structured MySQL relational database where the results of the different analyses are stored automatically by the pipeline. This database is key element for the pipeline, since the different analysis modules used in the pipeline get their input data from the database and write their output to it. The database contains information about all the data obtained, from cDNA libraries, isolated clones, and raw sequences to analysis results, as well as additional information about genetic maps and markers or expression data provided by users, or the representative clone selected for each unigene. It can also include information about the researchers and research centers involved, as well as journal publications or public accession numbers of sequences derived from the project. Access to the database can be password-protected in order to keep the data private.

### Web site

**Figure 4:**
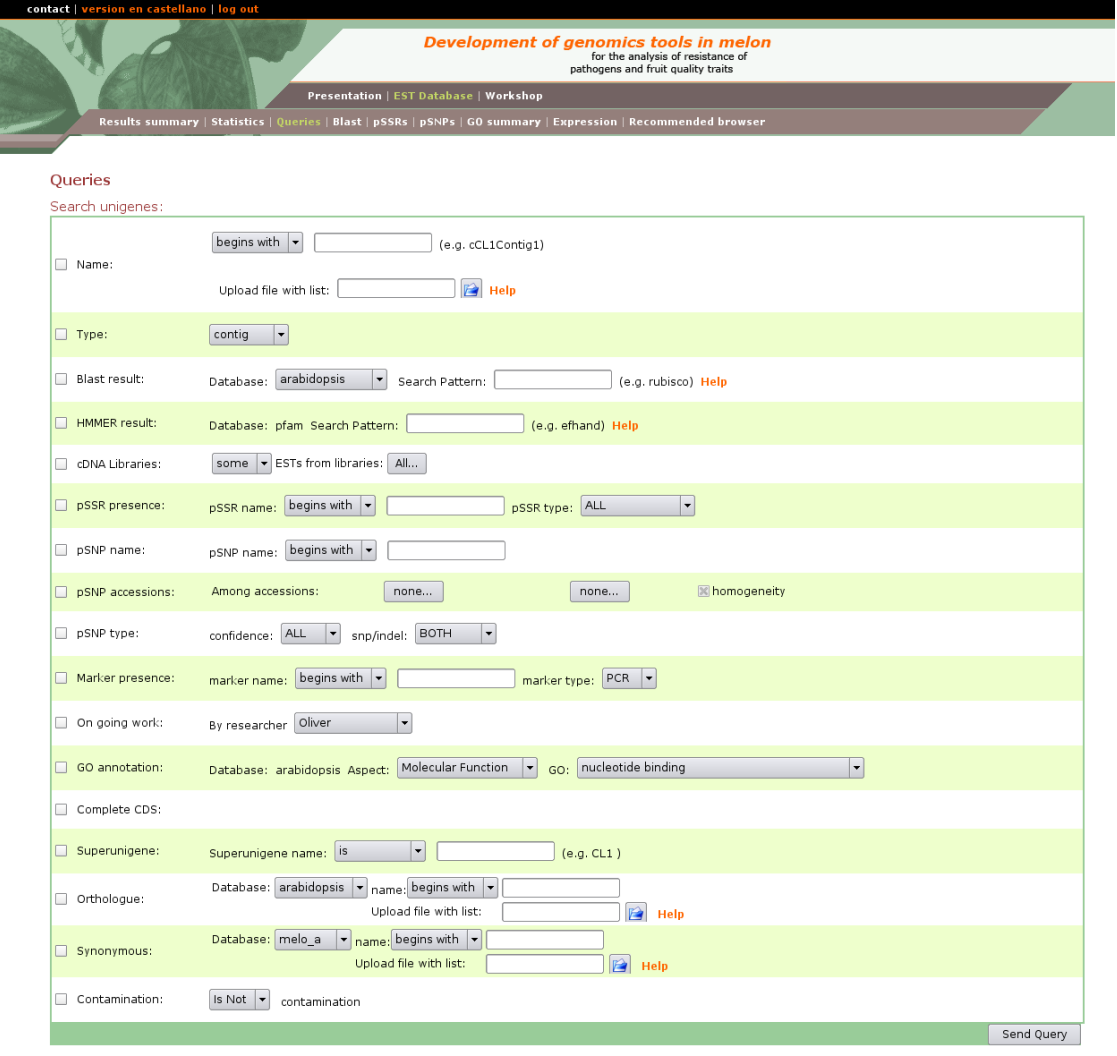
**A screenshot of the Queries page**. Unigenes can be efficiently retrieved by any combination of sequence features and/or annotations.

EST2uni includes a web site interface to the database through a powerful data mining tool with an advanced querying interface and high integration among all kinds of data (Figure 4). It is not just a collection of simple tables showing the data, or a simple query form to search by using sequence identifiers or keywords. On the contrary, it allows combination of almost every different functional and structural annotation criteria in the queries. For example, it would be very easy to look for unigenes fulfilling all the following criteria: 1) to have ESTs coming from several given libraries, 2) to be annotated as transcription factors, 3) to have sequences with tri-nucleotide SSRs or SNPs, and 4) to be represented by a putative full-length clone. Additionally, bulk queries using a file with a list of unigene names or orthologs are implemented.

**Figure 5:**
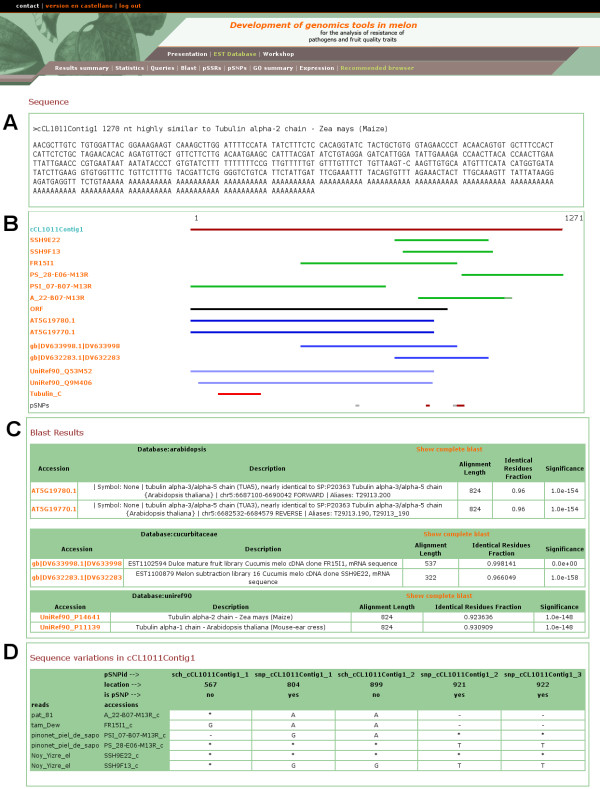
**A screenshot of a page with detailed information about a unigene**. (a) Unigene sequence and annotation. (b) Graphical representation of the unigene showing its nucleotide sequence, ESTs assembly, alignment to BLAST hits, predicted ORF-containing region, and functional and structural features. (c) Tables showing detailed information about the BLAST hits and hyperlinks to their respective web sites. (d) Table showing the nucleotide discrepancies among the ESTs in the unigene and their positions.

The unigenes obtained as query results can be inspected individually, but also bulk downloads of the sequences, names or orthologues are allowed. In order to globally analyze the Gene Ontology terms of the result set, it is possible to automatically submit the orthologues in some model species to Babelomics [16]. In a similar way, a text file with the expression data for the unigenes in the result set can be obtained, which can be used as a input for expression data analysis at specialized web sites like GEPAS [15], a well-known, web-based, microarray data analysis service. The individual unigene web page view shows graphical and textual summaries of the assembly and annotation results (Figure 5). Hyperlinks to the first hits of the external databases searched with BLAST are provided, as well as their descriptions and E values (Figure 5C). The full BLAST results can also be retrieved. Gene Ontology annotation results are also shown in a table with links to the GO-annotated similar external sequence used to transfer the GO annotation. Another table shows the sequence discrepancies among the ESTs in the unigene, so that putative SNPs can be evaluated by visual inspection (Figure 5D). Expression data for the unigene is also provided in this page. Finally, Primer3 [14] is integrated into the web site, and it is possible to design primers and see the graphical results with just two mouse clicks.

The web site also provides some statistics of the full EST collection, like contigs/singletons distribution, unigene length distribution, number of ESTs per unigene distribution, or number of unigenes annotated with the different functional and structural criteria. It also provides statistics for each single library, like number of ESTs, singletons, contigs, unigenes, novelty, and redundancy. Tables for global and library-specific Gene Ontology annotations are also provided where unigenes annotated in each functional category can be directly retrieved, and libraries can be compared for the Gene Ontology annotation of their unigenes.

### Comparison with other tools

Although other tools which perform EST analysis and database creation have been developed, none of them accomplishes all the characteristics we consider essential for the research community (Table 1). First, they must be freely available for download and local installation, as is the case for EST2uni. Some other tools (ParPEST [7], openSputnik [9], PipeOnline [2]), although promising, are not available for free download and local installation, and they must be used in collaboration with their maintainers under specific agreements. Second, they should be based, when possible, on freely-available, open-sourced, widely-used standard external tools. All the functionalities provided by EST2uni are based on such software tools, reducing its implementation costs and facilitating the incorporation of future improvements in these programs. This is not the case for some existing packages, which use proprietary tools like Oracle (ESTAP [3]) or RepeatBeater [37] (openSputnik [9]). Third, regarding the time efficiency of the software, only ParPEST [7] and EST2uni allow reduction of execution times by running the pipeline in a parallel environment. Fourth, EST2uni includes all the necessary steps for EST analysis, i.e., pre-processing, clustering and annotation, arranged in a sequential, completely automated pipeline. Similar existing tools provide at most different script programs (e.g., ESTAP [3], PartiGene [4]) and/or different interfaces (e.g., GARSA [8], PartiGene [4]) which need to be applied sequentially to perform the different steps in the standard EST analysis. Other tools (e.g., ESTIMA [5] and openSputnik [9]) provide only the database and its web interface (ESTIMA also provides scripts to load into the database the results of the EST analyses made by other means). Fifth, although all the similar existing tools perform some kind of functional annotation of EST collection, none allows structural annotation based on searching for SNPs and microsatellites, as does EST2uni. Sixth, only EST2uni provides a powerful data mining tool capable of complex queries by combining any structural and/or functional criteria to retrieve data. Similar existing tools only provide static tables showing different statistics related to the EST collection, and simple web forms to retrieve data with simple queries, such as EST/unigene ID or length, and BLAST hit description keywords. Lastly, none of the other tools integrate expression data storage. To sum up, the EST2uni package is, as far as we know, the only application able to meet all the requirements of the research community.

**Table 1:** Comparison with similar tools. Features of different EST analysis and database tools.

	PartiGene	ParPEST	openSputnik	ESTAP	ESTIMA	GARSA	PipeOnline	EST-PAGE	EST2uni
freely available^1^	X			X	X	X		X	X
based in non-comercial tools	X	X			X	X	X	X	X
parallelized		X							X
expression data integration									X
completely automated		X					X		X
complex data mining^2^									X
SNPs identification									X
genetic markers integration									X
microsatellites identification			X						X
orthologues identification									X
GO annotation		X	X		X			X	X
functional motifs identification			X			X			X

^1^It means that software package can be free and directly downloaded from Internet for local installation and use.

^2^It means the ability to perform complex queries to the database using any combination of EST/unigene annotations as a condition in the query clause.

## Conclusion

We have developed an EST analysis tool capable of converting, in a fully automatic and time-effective way, a set of trace files or plain sequences in a highly structured and annotated EST database with a user-oriented web interface for efficient data mining. The EST analysis pipeline includes standard pre-processing, clustering and annotation programs, and can incorporate gene expression data to the database. The software is highly modular, which facilitates the incorporation of new methods and analyses, meeting the needs of different EST projects. Running options are also easily adapted to local needs by simply modifying an extensively documented single configuration text file which provides the parameters to be used by the different analyses. Once configured, the pipeline runs without user assistance, from the input files to the final annotated EST database. The EST analysis can be run either in a single standard computer, or in a PC-cluster, thus taking advantage of the multiprocessing capabilities of these systems, which allows reduction of the time required to complete the EST analysis. The web site deployed is a powerful data mining tool with a complex, yet easy to use, query interface, that also provides functionalities for bulk data retrieval and download. It also eases the use of several tools, like primer design and BLAST searches against the database. Access to the data can be restricted by passwords to keep the data private. The development team of EST2uni is continuously improving the end-user interface, the quality of the analyses, and the integration with other tools. We have set up a public subversion server [38], and a mailing list [39] to allow collective development of the code, which everyone is invited to join.

To sum up, we conceive this bioinformatics tool as an open and evolving project, and all the bioinformatics community is invited to participate, using and improving the tool that we have created for our specific EST projects (Citrus Functional Genomics Project [10], ChillPeach Project [11], and Spanish Melon Genomics Project [12]).

## Availability and requirements

EST2uni can be freely downloaded from Internet [17]. Detailed installation instructions are provided on the download web page and are also included in the software package. The application is free in the sense that it has been released under the GPL license, and its development is open and collaborative. Any researcher is free to use it, to modify it, and to deploy their own web site with the results.

No hardware or memory restrictions are imposed by this software package other than those that apply to the external programs used in the pipeline. In general, it runs without problems in any standard medium-level ix86-based equipment using the Linux operating system. We have tested and configured correctly this software in computers running the following GNU/Linux distributions: Fedora Core 5, Ubuntu 6.06, SuSE Linux 8.2, and Debian Sarge. It should run without problems in any Unix installation as long as all the required software is installed. The following software, which come with any GNU/Linux distribution, is absolutely required to run the EST2uni pipeline: Perl, Apache, MySQL, and PHP. EST2uni also requires certain additional Perl modules, specifically Bioperl and DBI. In addition, the following external tools and resources are used to perform the different analysis: phred, Lucy, RepeatMasker, Repbase, SeqClean, NCBI's UniVec, TGICL, ESTScan, sputnik, NCBI BLAST, ipcress, and the Perl module go-perl. For parallel processing, the load balancing tool Condor must be installed. Detailed instructions for installing each of these external programs are also provided with the package and at the EST2uni download web site.

## Competing interests

The author(s) declares that there are no competing interests.

## Authors' contributions

JMB and JF conceived and designed the pipeline structure, the database schema, and the web site, wrote the code, tested EST2uni as end-users, and drafted the manuscript. FG wrote the code for parallelization, tested its efficiency, and drafted the parallelization section of the manuscript. AR managed the parallelization project, and critically revised the manuscript. VC and FN critically revised the manuscript, and coordinated the Citrus Functional Genomics Project and the Spanish Melon Genomics Project, respectively, which provided the EST data which were utilized in the development and testing of EST2uni. JMB guided and coordinated the development of EST2uni. All authors read and approved the final manuscript.
